# Collecting sensitive information for a sexual health trial with young people: experiences of using electronic data collection and traditional paper methods

**DOI:** 10.1186/1745-6215-16-S2-P18

**Published:** 2015-11-16

**Authors:** Lisa Maguire, Aine Aventin, Dirk Schubotz, Laura Dunne, Maria Lohan, Mike Clarke

**Affiliations:** 1 Queen's University Belfast, Belfast, UK; 2 University of Liverpool, Liverpool, UK

## 

Electronic data collection for randomised trials is attractive as it can, in theory, provide great financial advantages by reducing fieldwork costs and data inputting time, as well as increasing the accuracy of responses over and above paper based methods. For trials involving young people, electronic methods provide other advantages such as the ability to adapt questions to meet the needs of young people of differing ages and abilities. Researchers have also suggested that young people prefer electronic methods over traditional ones and have found equal validity across both methods. This presentation will discuss issues relating to the collection of sensitive information from young people in Northern Ireland, using electronic and paper formats. We will draw on experience gained across several research studies that have collected data on sexual risks and behaviours, particularly in school-based research; including a recent trial on adolescent pregnancy. We will examine the feasibility, reliability and validity of data collected along with an evaluation of the cost implications and ease of using both methods (including the merging of the resulting datasets if both methods are offered). The views of the researchers and participants will also be reflected upon.

